# Estimating the synaptic density deficit in Alzheimer’s disease using multi-contrast CEST imaging

**DOI:** 10.1371/journal.pone.0299961

**Published:** 2024-03-14

**Authors:** Syed Salman Shahid, Mario Dzemidzic, Elizabeth R. Butch, Erin E. Jarvis, Scott E. Snyder, Yu-Chien Wu

**Affiliations:** 1 Department of Radiology and Imaging Sciences, Indiana University School of Medicine, Indianapolis, IN, United States of America; 2 Department of Neurology, Indiana University School of Medicine, Indianapolis, IN, United States of America; 3 Stark Neurosciences Research Institute, Indiana University School of Medicine, Indianapolis, IN, United States of America; 4 Weldon School of Biomedical Engineering at Purdue University, West Lafayette, IN, United States of America; University Tunku Abdul Rahman, MALAYSIA

## Abstract

In vivo noninvasive imaging of neurometabolites is crucial to improve our understanding of the underlying pathophysiological mechanism in neurodegenerative diseases. Abnormal changes in synaptic organization leading to synaptic degradation and neuronal loss is considered as one of the primary factors driving Alzheimer’s disease pathology. Magnetic resonance based molecular imaging techniques such as chemical exchange saturation transfer (CEST) and magnetic resonance spectroscopy (MRS) can provide neurometabolite specific information which may relate to underlying pathological and compensatory mechanisms. In this study, CEST and short echo time single voxel MRS was performed to evaluate the sensitivity of cerebral metabolites to beta-amyloid (Aβ) induced synaptic deficit in the hippocampus of a mouse model of Alzheimer’s disease. The CEST based spectra (Z-spectra) were acquired on a 9.4 Tesla small animal MR imaging system with two radiofrequency (RF) saturation amplitudes (1.47 μT and 5.9 μT) to obtain creatine-weighted and glutamate-weighted CEST contrasts, respectively. Multi-pool Lorentzian fitting and quantitative T1 longitudinal relaxation maps were used to obtain metabolic specific apparent exchange-dependent relaxation (AREX) maps. Short echo time (TE = 12 ms) single voxel MRS was acquired to quantify multiple neurometabolites from the right hippocampus region. AREX contrasts and MRS based metabolite concentration levels were examined in the ARTE10 animal model for Alzheimer’s disease and their wild type (WT) littermate counterparts (age = 10 months). Using MRS voxel as a region of interest, group-wise analysis showed significant reduction in Glu-AREX and Cr-AREX in ARTE10, compared to WT animals. The MRS based results in the ARTE10 mice showed significant decrease in glutamate (Glu) and glutamate-total creatine (Glu/tCr) ratio, compared to WT animals. The MRS results also showed significant increase in total creatine (tCr), phosphocreatine (PCr) and glutathione (GSH) concentration levels in ARTE10, compared to WT animals. In the same ROI, Glu-AREX and Cr-AREX demonstrated positive associations with Glu/tCr ratio. These results indicate the involvement of neurotransmitter metabolites and energy metabolism in Aβ-mediated synaptic degradation in the hippocampus region. The study also highlights the feasibility of CEST and MRS to identify and track multiple competing and compensatory mechanisms involved in heterogeneous pathophysiology of Alzheimer’s disease in vivo.

## Introduction

Alzheimer’s disease (AD) is a progressive neurodegeneration disorder and the most common type of dementia. The neuropathological hallmarks of AD include abnormal accumulation of beta-amyloid (Aβ) in extracellular neuritic plaques and phosphorated tau proteins in intra-cellular neurofibrillary tangles (NfTs) [1,2]. Abnormal changes in synaptic organization (synaptic density) and associated impaired synaptic transmission (neuronal connectivity) have shown to be associated with neurodegenerative diseases [3–6]. Recent studies suggest that alterations in dendritic arborization could be one of the primary causes for the synaptic and neuronal loss in AD [7]. In a mouse model of AD dendritic deficits appeared before quantifiable accumulation of Aβ and those observed early synaptic changes correlated with cognitive performance scores [8].

In pathology associated brain aging, significant physiological changes such as dendritic deficit, neuronal loss, impaired cerebrovasculature and elevated inflammation lead to abnormal functional and behavior changes. These physiological factors directly impact neurochemistry. Therefore, alterations of the cerebral metabolite concentration levels may be informative about these physiological changes. Although the mechanisms involving synaptic deficit in AD are still inconclusive, one of the hypotheses involves excessive and dysregulated glutamate (Glu) neurotransmission and excitotoxicity [9]. Alterations in Glu levels in the presence of pathology may indicate loss of glutamatergic neurons possibly due to the disturbance of Glu synthesis and/or dysfunction in glutamate/glutamine recycling mechanism between astrocytes and neurons [10].

Impairment in synaptic density due to mitochondrial perturbation has recently been proposed as an early indicator of AD-associated neurodegeneration [11,12]. This mitochondrial cascade hypothesis may provide an alternate or complementary perspective on the pathogenesis of AD [13]. Data from studies using postmortem AD brains and AD animal models highlight the importance of mitochondria and their energy metabolism in synaptic transmission and synaptic health in AD pathology [14]. Creatine-phosphocreatine system (creatine kinase reaction: CKR) plays an important role in cerebral energy metabolism [15]. Quantifying in vivo cerebral creatine (Cr) may thus reveal bioenergetic abnormalities in synaptic impairment [16].

Synaptic density and synaptic function have been extensively studied in various animal models of neurodegeneration with Positron emission tomography (PET) [17–20]. Despite the molecular accuracy of PET, several factors (high operational cost, low spatial resolution, unspecific binding of the radio tracers outside the brain, exposure to ionizing radiation) restrict its wider use. Magnetic resonance (MR) imaging on the other hand, is a safe, non-invasive, and non-irradiating imaging technique. MR-based molecular imaging methods, magnetic resonance spectroscopy (MRS) and chemical exchange saturation transfer (CEST), can quantify multiple metabolic functions and may provide promising endogenous contrast-based indices of synaptic health [21–24].

In this study, we used Glu-weighted and Cr-weighted endogenous CEST contrasts to map the synaptic deficit in the ARTE10 mouse model of AD [25]. In conjunction with ^1^H-MRS and/or immunohistochemistry, Glu-CEST has previously been used in vitro and in vivo preclinical models of neurodegeneration [26–34]. As MR-based Glu signal is derived from multiple compartments, including cytosol, synaptic vesicles, and extracellular matrix [35,36], these compartments may contribute differently to MR-measured Glu depending on the acquisition parameters. It has been suggested that 20–30% of Glu is not MR-visible at echo times (TE) ≥ 20ms and that this invisible Glu may be the part of neurotransmitter pool (mitochondrial or synaptic vesicles) [37–39]. To account for this source of variability in Glu quantification, we used a short echo time (TE = 12ms) Semi-Localization through Adiabatic Selective Refocusing (semi-LASER) single voxel MRS sequence. We used quantitative maps of longitudinal relaxation time (qT1) and tissue volume segmentation (fraction) to first perform MRS-based absolute Glu quantification and then assessed its association with Glu-CEST contrast. Additionally, we wanted to assess the role of Aβ mediated reactive oxygen species and the impact of oxidative stress on mitochondrial dysfunction and impaired energy metabolism. To accomplish that, we also acquired Cr-CEST contrast and estimated its contribution to MRS based creatine using linear association analysis.

We hypothesize that multi-modal quantitative MR approaches can be an effective in vivo tool to probe multiple competing and compensatory mechanisms involved in Aβ-induced synaptic degradation. The focus of this study is on glutamatergic neuron rich hippocampus region, as abnormal depositions of Aβ and NfTs have previously been reported in the medial temporal lobe, including the hippocampus during the early stages of AD [25,40]. In this exploratory study, we aimed to assess the feasibility of in vivo quantification of neurochemical variations associated with synaptic degradation in the hippocampus of an AD mouse model. Our methodology involved the utilization of multiple CEST contrasts, highly resolved single voxel spectra (SVS) ^1^H-MRS, and advanced MRI/MRS processing techniques. Through this approach, we aim to gain insights into the pathophysiological mechanisms associated with synaptic degradation in the hippocampus during the progression of AD.

## Material and methods

### Numerical simulation

**Fig 1:**
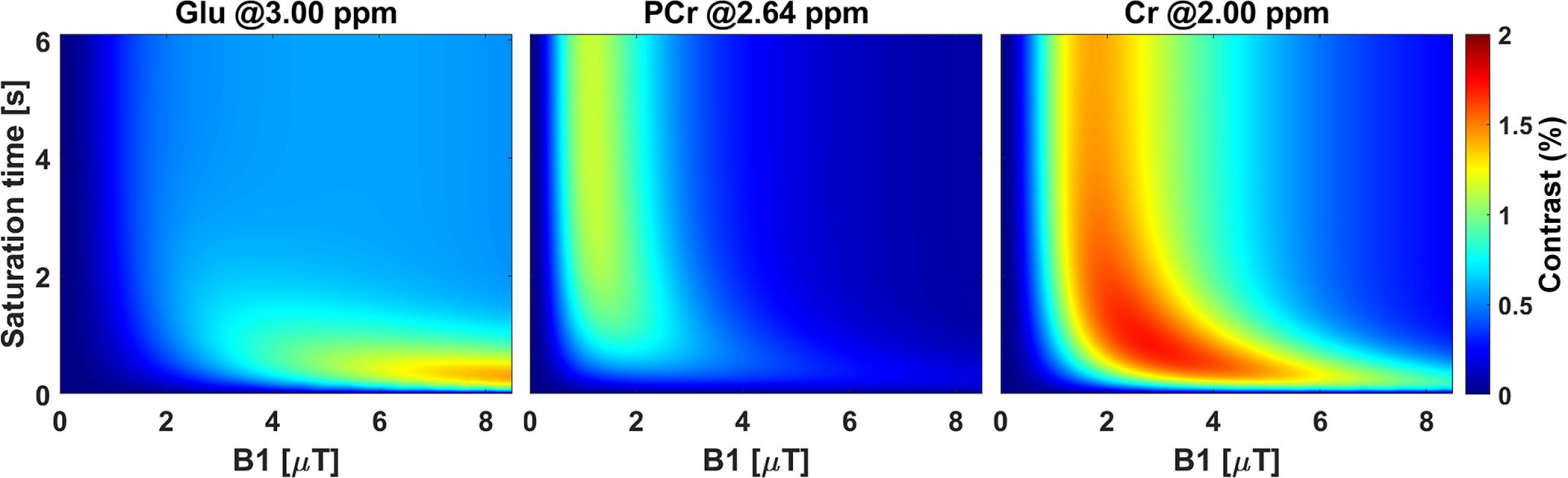
Numerical simulation of CEST contrasts as a function of B1 amplitude and saturation duration at 9.4 T. The Glu-CEST contrast is shown at 3.00 ppm, PCr-CEST contrast at 2.64 ppm and Cr-CEST contrast at 2.0 ppm. The simulation parameters of the multi-pool model were obtained from the literature [41–44]. Each metabolite distribution map was obtained by taking the Z-spectra difference at the frequency of the metabolite of interest with and without the respective pool. ppm = parts per million.

To investigate molecular specificity and enhance the detection sensitivity of the exchange species of interest at 9.4 T, we initially used numerical simulation based on modified Bloch-McConnell equation for a 9-pool model [41]. The model contained water pool at 0 ppm, fast exchanging amine solute pool at 3 ppm, symmetric semisolid component pool centered at 0 ppm or asymmetric semisolid component pool centered at -2.3 ppm, guanidinium protons of Cr and PCr at 2.0 ppm and 2.64 ppm, respectively, Amide protons at 3.5 ppm and nuclear overhauser enhancement (NOE) at -3.5 and -1.6 ppm, respectively. These simulation parameters of the multi-pool model were obtained from the literature [41–44]. For the simulation, the block pulse saturation amplitude was varied from 0.5–8.5 μT and for each experiment, the saturation pulse duration was varied from 500 ms– 6100 ms. The repetition time for each simulation was set at 8000 ms. For each simulation, CEST based spectra (Z-spectra) were generated with saturation offset from –8 ppm to 8 ppm with 0.08 ppm increment. Each metabolite distribution map was obtained by taking the Z-spectra difference at the frequency of the metabolite of interest with and without the respective pool. In each model the effect of symmetric and asymmetric semisolid magnetization transfer (MT) pool was separately simulated (Fig 1).

### Animal model and animal preparation

All animal care and research were performed in accordance with the National Institutes of Health guidelines. All study procedures were approved by the Indiana University School of Medicine Institutional Animal Care and Use Committee. The MR experiments were performed on double transgenic mouse model of Alzheimer’s disease harboring mutant forms of human Aβ precursor protein (APP) and Presenilin-1 transgenes [25]. These ARTE10 animals (B6.CBA-Tg (Thy1-PSEN1*M146V, -APP*Swe)) were obtained from Taconic Biosciences Inc (Germantown, NY, USA). At 3 months of age, the ARTE10 animals exhibit AD-like amyloid pathology in the subiculum and anterior neocortex, which progresses further and encroaches to other regions with age [45]. These animals do not exhibit tau pathology up to 20 months [45]. Compared to wild type (WT, C57BL/6NT) littermates, these animals do not exhibit neuronal loss, however, 20% reduction in dendritic arbor has been reported in the hippocampus at 12 months [45,46]. Thus, ARTE10 serves as a good model of Aβ associated synaptic density deficit without the confounding effects of tau pathology and neuronal loss [25].

For MRI experiments, the animals were initially anesthetized in an induction chamber under 3% isoflurane at 1 liter per minute in 100% oxygen. The anesthetized mice were then transferred to an MR compatible cradle and positioned in an MRI compatible head holder to minimize head motion. Anesthesia was subsequently maintained at 1.5% isoflurane in 100% oxygen throughout imaging. Respiration rate was monitored using a pressure pad placed under the animal abdomen and animal body temperature was maintained by a warming pad (37°C) placed under the animal. Respiration rate was maintained between 80 and 125 breaths per minute using manual adjustments to isoflurane vaporizer.

### MR acquisition

**Fig 2:**
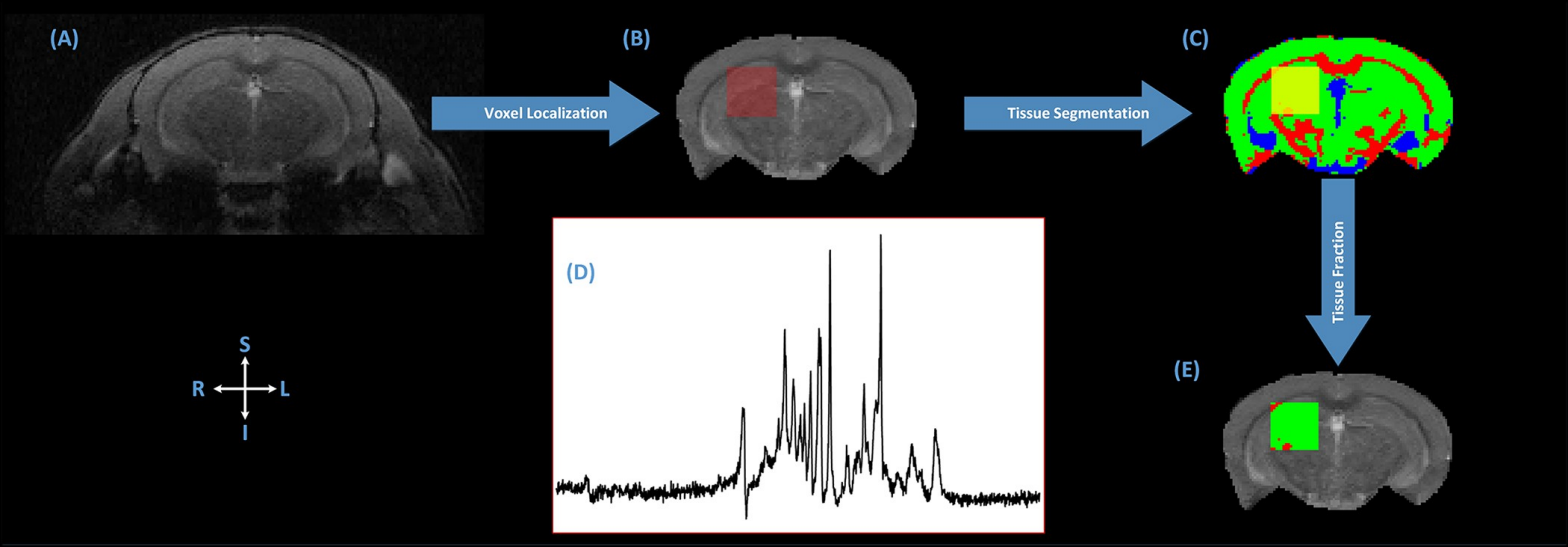
Schematic representation of the MRI/MRS fusion framework used in the analysis. (A) a high-resolution T2-weighted anatomical reference image was used for the manual placement of a single coronal (1 mm^2^ thick) slice for qT1 and CEST imaging covering the hippocampus region, and a 2 mm^3^ voxel in the right hippocampus region of the animal for single voxel ^1^H-MRS. (B) Skull stripped, denoised and B1 bias field corrected T2-weighted reference image with ^1^H-MRS voxel. (C) Gray matter (GM), white matter (WM) and cerebrospinal fluid (CSF) classification using the corrected T2-weighted anatomical scan. (D) Raw water-suppressed ^1^H-MR spectra from the prescribed voxel location. (E) Tissue volume fractions in MRS voxel only. L and R indicate anatomical orientation (left and right). S and I indicate anatomical orientation (superior and inferior).

The in vivo imaging was conducted on a horizontal bore 9.4 T Biospec pre-clinical MRI system (Bruker BioSpin MRI GmbH, Germany) equipped with shielded gradients (maximum gradient strength = 660 mT/m, rise time = 4750 T/m/s). We used an 86 mm quadrature volume resonator for transmission and a 4-element array cryo-coil for signal reception (cryoprobe, Bruker, BioSpin). A high-resolution FLASH based multi-slice 2D anatomical reference image facilitated single slice CEST and MRS voxel (2.0 mm^3^) placement in the right hippocampus region (Fig 2). The reference scan acquisition parameters were TE/TR = 3/15 ms, slice thickness = 500 μm, acquisition matrix = 192×192, field of view (FoV) = 20x20 mm^2^, flip angle (FA) = 10°, in-plane voxel resolution = 78x78 μm^2^, number of slices = 20, number of averages = 3, and 3 slice packages.

For CEST imaging, a single 1-mm thick coronal slice covered the hippocampus region as guided by the anatomical reference image. Glu-CEST was acquired using a turbo-rapid acquisition relaxation enhancement (RARE) sequence with the following acquisition parameters: TE/TR = 5.15/5000ms, FoV = 16x16 mm^2^, acquisition matrix = 128x128, in-plane voxel resolution = 125x125 μm^2^, slice thickness = 1.0 mm, averages = 1, RARE factor = 64, phase encoding order = centric, fat suppression = on. 57 saturation frequency offsets (−8.0 ppm to 8.0 ppm) and a reference offset image at −300 ppm were acquired using a continuous-wave (CW) radiofrequency (RF) saturation pulse (B1_sat_ = 5.9 μT; block pulse shape). For Glu-CEST acquisition, the duration of the saturation pulse was 1000 ms. For Cr-CEST acquisition, most of the acquisition parameters were similar to the Glu-CEST sequence, except the following: CW RF saturation pulse power (B1_sat_ = 1.4 μT). The duration of the B1_sat_ pulse was 3500 ms. 83 asymmetric saturation frequency offsets ranging from −8 ppm to 8 ppm with a reference offset (non-saturated) image at −300 ppm was used (−8 ppm to 0 ppm, Δω = 0.25 ppm; 0.05 ppm to 2.75 ppm, Δω = 0.1 ppm; 2.8 ppm to 4.3 ppm, Δω = 0.125 ppm; 4.4 ppm to 6 ppm, Δω = 0.2 ppm; and 6.5 ppm to 8 ppm, Δω = 0.5 ppm).

Static magnetic field (B0) map was acquired to correct for local B0 field inhomogeneity in Glu-CEST and Cr-CEST acquisitions using water saturation shift referencing (WASSR) acquisition [47]. For WASSR acquisition, 81 saturation frequency offsets were acquired with B1_sat_ = 0.05 μT, saturation pulse duration = 1000 ms and saturation offset ranging from −1 ppm to 1 ppm with 0.025 ppm increment. RF field (B1) inhomogeneity was corrected using double angle method [33,48]. Two GRE acquisitions with the following parameters were used to obtain B1 transmit (B1^t^) map. TE/TR = 3.32/15000 ms, flip angles = 30° and 60°. Preparation pulse shape = block, pulse duration = 42.67 μs. FoV = 16x16 and acquisition matrix = 64x64. To account for T1 longitudinal relaxation on CEST contrasts, a T1 map using RAREVTR (RARE with variable TR) sequence was used. T1map acquisition has identical geometrical setup as that of CEST acquisition, with the following parameters: TE = 6.11ms, T_I_ = [0.25, 0.5, 1.0, 1.5, 2.0, 3.5, 5.0, 8.0] sec, RARE factor = 4 and single average.

^1^H-MRS spectra were acquired from a 2mm^3^ voxel localized in the right hippocampus region (Fig 2). The MRS voxel was localized for all study animals, but due to time constraints actual single voxel spectra were acquired for *n* = 7 animals per group using semi-LASER sequence [49,50]. Before the spectra acquisition, automated localized shimming was performed using Bruker provided FASTMAP method. The field was shimmed to < 17.30 Hz full width at a half maximum (FWHM) linewidth of the unsuppressed water peak. The following parameters were used for spectra acquisition: TE/TR = 12/2500 ms, 4096 complex data points, spectral width = 8000 Hz, outer volume suppression (OVS) enabled, and water signal suppression was achieved using variable power and optimized relaxation delays (VAPOR) scheme. VAPOR pulse amplitudes were adjusted manually for each animal to achieve optimal water suppression. OVS slice thickness = 5mm, gap to voxel = 1 mm, number of dummy scans = 8, number of averages = 128. The slice selective 90° RF pulse followed by two pairs of slice-selective adiabatic refocusing pulses in other two dimensions were offset by Δf = −2.3 ppm to minimize the chemical shift displacement artifacts in quantifying metabolite concentrations. An additional SVS was acquired as a reference for absolute metabolic quantification using the same acquisition parameters as above except that water suppression was not enabled. The number of averages for unsuppressed spectra was 8.

### MR data processing

**Fig 3:**
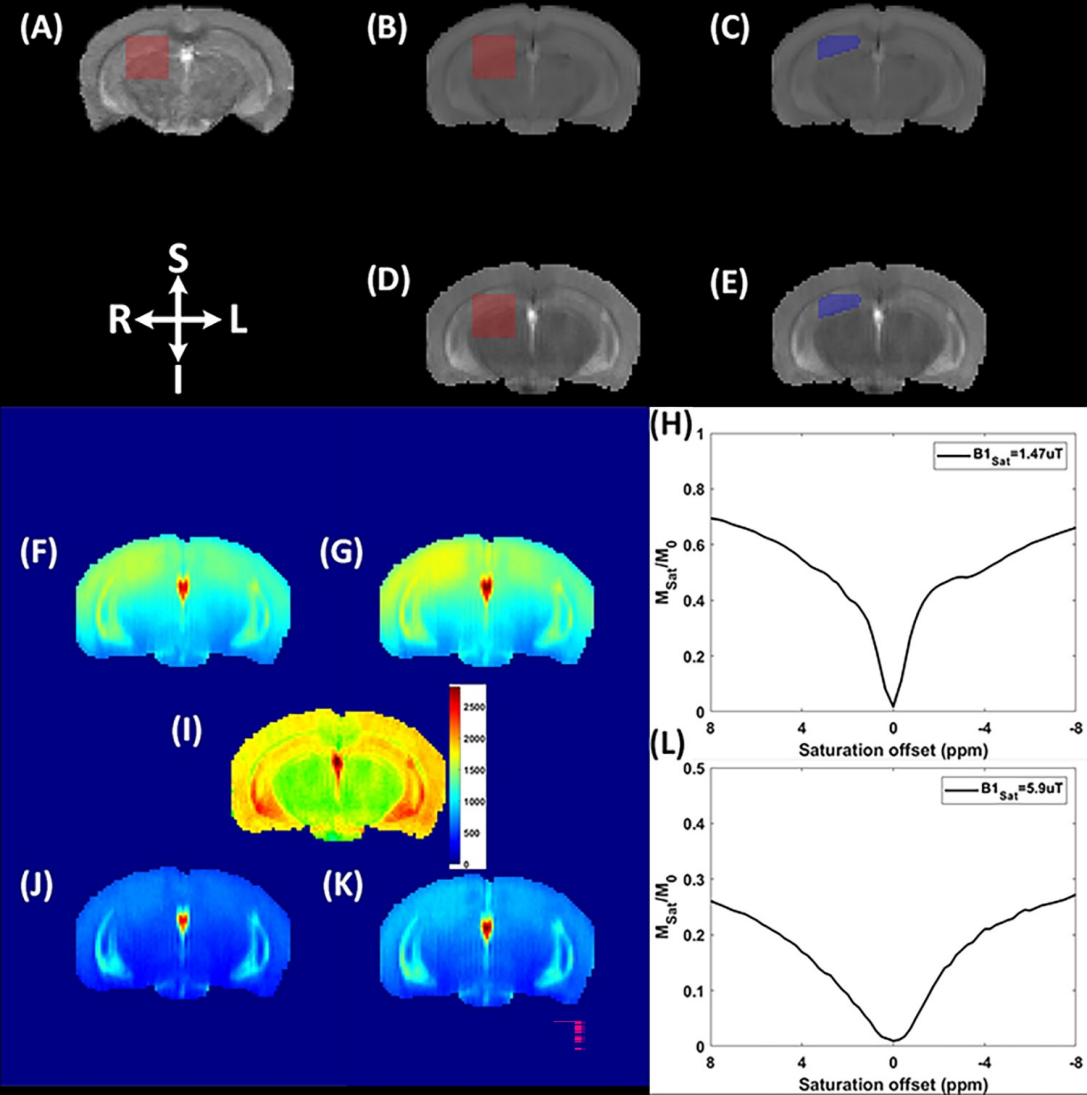
Qualitative and quantitative maps from a representative WT animal. (A) Coronal view of the anatomical reference image and ^1^H-MRS voxel registered to qT1 map. (B) T_1_-weighted image with translated ^1^H-MRS voxel. (C) T_1_-weighted image with hippocampus section within the ^1^H-MRS voxel. (D) qT1map with translated ^1^H-MRS voxel. (E) qT1map with hippocampus section within the ^1^H-MRS voxel. (F) Normalized CEST image at Δω = 3 ppm using B1_sat_ = 5.9 μT. (G) Normalized CEST image at Δω = −3 ppm using B1_sat_ = 5.9 μT. Normalized Z-spectra from the right hippocampus ROI using B1_sat_ = 1.47uT. (I) qT1map in false RGB colors and range scaled to 2700 ms to highlight various tissue types. (J) Normalized CEST image at Δω = 2 ppm using B1_sat_ = 1.47 μT. (K) Normalized CEST image at Δω = −2 ppm using B1_sat_ = 1.47 μT. (L) Normalized Z-spectra from the right hippocampus (ROI) using B1_sat_ = 5.9 uT. L and R indicate anatomical orientation (left and right). S and I indicate anatomical orientation (superior and inferior).

The high-resolution anatomical reference image was first denoised using Non-local Means (NLMeans) filter implemented in ANIMA (https://anima.irisa.fr) [51] and then skull stripped [51], and bias field corrected [52]. Using the corrected reference image, three class (gray matter (GM), white matter (WM), and cerebrospinal fluid (CSF)) tissue segmentation was performed using ANTs Atropos, an ITK-based multi-class brain segmentation method [53]. A custom-build python script was used to map the MRS voxel location to the reference image (Fig 2). Separately, hippocampus region was segmented using the procedure described in [54]. The tissue volume fractions within the MRS voxel were quantified using the tissue specific binary masks (see Fig 2C and 2E) that were later used in absolute metabolite quantification [55]. Quantitative T1map (qT1map) from the single slice RAREVTR acquisition was obtained using Paravision-360 ‘*Image sequence analysis*’ tool with the default settings. qT1map and T_I_ weighted images were skull stripped and then linearly registered to the anatomical reference image (Fig 3). The resulting transformation matrix was used to translate MRS voxel map, bilateral hippocampus mask, GM, WM and CSF masks, and their respective volume fractions to qT1/CEST space (Fig 3A–3E).

For CEST data, voxel-by-voxel Z-spectra were generated by mapping the longitudinal magnetization as a function of saturation frequency (Fig 3H and 3L). For CEST processing, the initial step included B0 field inhomogeneity correction using the water shift referencing method [47]. The B0-corrected CEST data were then normalized using the reference (non-saturated; −300 ppm) image. To improve Glu-CEST contrast at Δω = 3 ppm, we applied symmetric magnetization transfer (MT) baseline removal and apparent exchange dependent relaxation (AREX) based quantification. Initially, a 3-pool Lorentzian model consisting of MT, direct saturation (DS), and nuclear Overhauser enhancement (NOE_-3.5ppm_) was fitted to the B0 corrected normalized Z-spectra in a voxel-by-voxel manner. The Z-spectra was fitted as a sum of multiple Lorentzian line shapes using the following equation:

$$1=\frac{I}{I_{0}}-\overset{N}{\underset{i=1}{\sum }}[\frac{A_{i}}{1+4{\left(\frac{\Delta \omega -\Delta \omega_{i}}{\sigma_{i}}\right)}^{2}}]$$ (1)

Here, *Δω* is the frequency offset in ppm with respect to water resonance of 0 ppm. *A*_*i*_, *Δω*_*i*_ and *σ*_*i*_ are the amplitude, frequency offset, and the linewidth of the CEST peak of the *i*^*th*^ proton pool, respectively. The chemical shift frequencies for each proton pool, the initial conditions, and the upper and lower bounds of the fits of respective species’ amplitude and linewidth parameters are described in Table 1. The Z-spectra fitting was performed in Matlab using the non-linear fitting ‘*lsqnonlin’* function and the processing code adapted from [56]. To remove the MT baseline and DS effects, we applied inverse subtraction method:

$$\frac{1}{Z_{CORR}}=\frac{1}{Z(\Delta \omega )}-\frac{1}{Z_{FIT_{MT}}(\Delta \omega )}$$ (2)

**Table 1:** Starting points and boundaries of the amplitude (A), peak width (*σ* in ppm) and frequency offset (Δω in ppm) of the coupling pools in the Lorentzian fit. The values were taken from [42,57–60].

Pool	A	σ [ppm]	Δω [ppm]
	LB/UB/SV	LB/UB/SV	LB/UB/SV
Water	0.02/1/0.9	0.3/10/1.4	−1/1/0
NOE_-3.5_	0/0.6/0.02	0.5/10/3	−4/0/−2
NOE_-1.6_	0/0.2/0.001	0/1.5/1	−2/−1/−1.5
MT_symmetric_	0/1/0.1	10/100/25	−4/4/0
MT_asymmetric_	0/1/0.1	10/100/25	−4/4/−2

Abbreviations: LB: Lower bound, UB: Upper bound, SV: Starting value, NOE: Nuclear Overhauser enhancement, MT: Magnetization transfer.

The use of AREX method to Z_CORR_ data, mitigated the T1 effects:

$$AREX_{CORR}=(\frac{1}{Z_{CORR}(\Delta \omega )}-\frac{1}{Z_{CORR}(-\Delta \omega )})\frac{1}{T_{1}}$$ (3)

**Fig 4:**
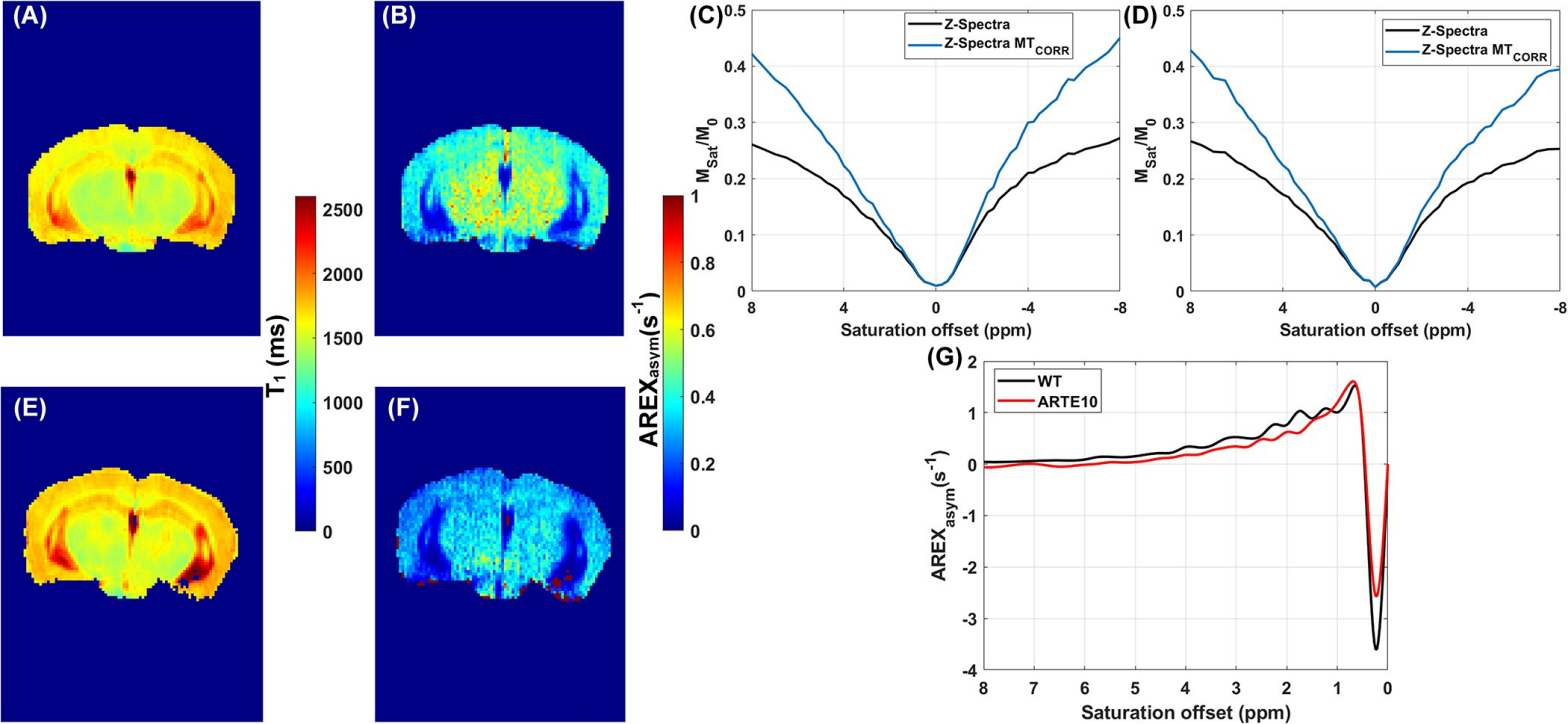
Quantitative maps from the representative animals. (A) Coronal slice of a qT1 map from a representative WT (control) animal. (B) Glu-AREX map (B1_sat_ = 5.9 μT; Δω = 3 ppm) from the same WT animal. (C) Normalized mean Z-spectra and normalized mean MT baseline corrected Z-spectra from the right hippocampus region of the representative WT animal. (D) Normalized mean Z-spectra and normalized mean MT baseline corrected Z-spectra from the right hippocampus region of the representative ARTE10 animal. (E) Coronal slice of a qT1 map from a representative ARTE10 animal. (F) Glu-AREX map (B1_sat_ = 5.9 μT; Δω = 3 ppm) from the same ARTE10 animal. (G) Mean AREX asymmetry spectra from the right hippocampi of the representative WT (black line) and ARTE10 (red line) animals.

**Fig 5:**
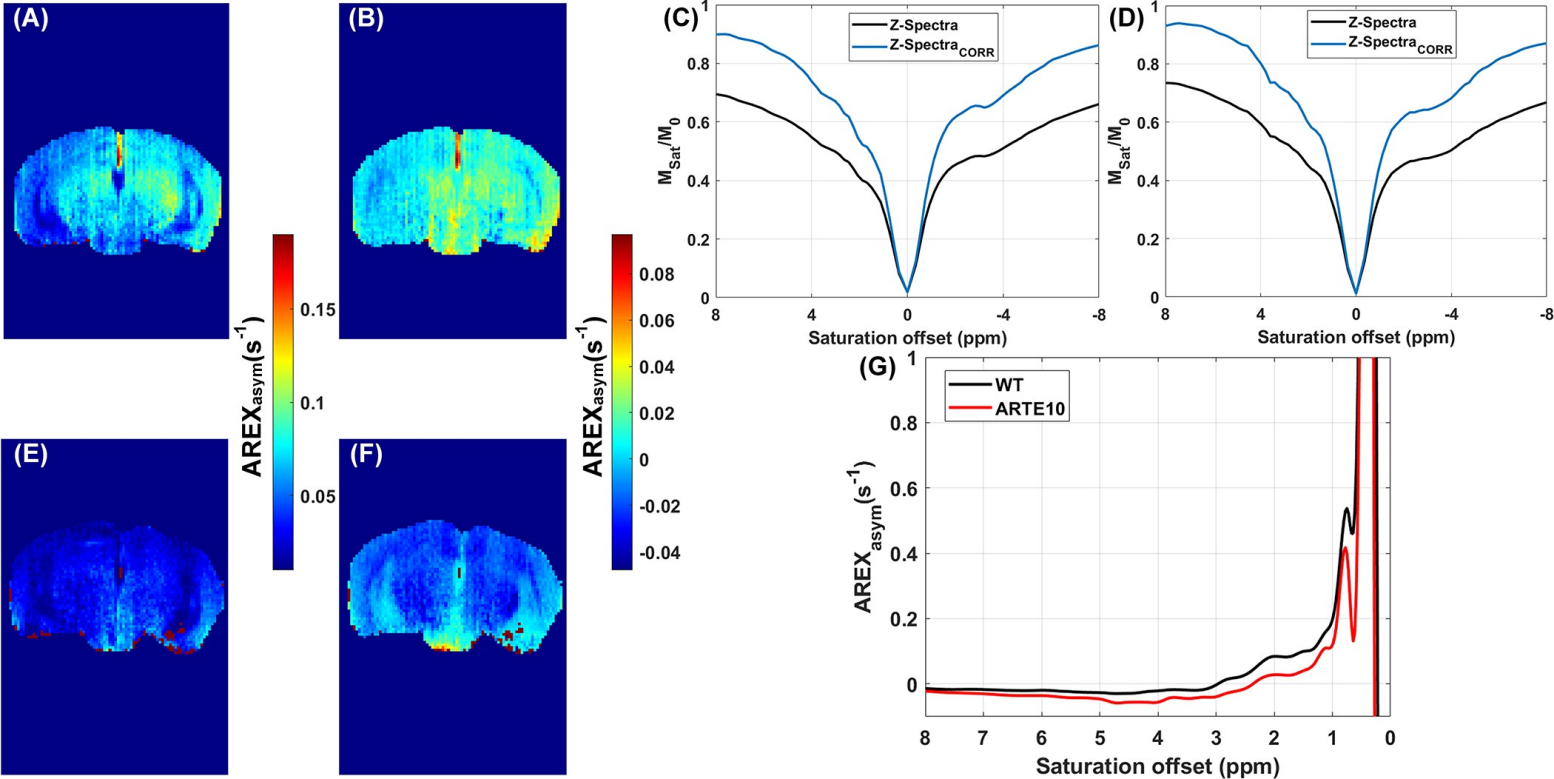
Quantitative CEST derived maps from the representative animals. (A) Cr-AREX map (B1_sat_ = 1.47 μT; Δω = 2 ppm) from a representative WT (control) animal. (B) PCr-AREX map (B1_sat_ = 1.47 μT; Δω = 2.64 ppm) from the same WT animal. (C) Normalized mean Z-spectra and normalized mean MT baseline corrected Z-spectra from the right hippocampus region of the representative WT animal. (D) Normalized mean Z-spectra and normalized mean MT baseline corrected Z-spectra from the right hippocampus region of the representative ARTE10 animal. (E) Cr-AREX map (B1_sat_ = 1.47 μT; Δω = 2 ppm) from a representative ARTE10 animal. (F) PCr-AREX map (B1_sat_ = 1.47 μT; Δω = 2.66 ppm) from the same ARTE10 animal. (G) Mean AREX asymmetry spectra from the right hippocampi of the representative WT (black line) and ARTE10 (red line) animals.

For Glu-based AREX contrast, *Δω* = 3 ppm (Fig 4B and 4F). For Cr- and PCr-CEST based processing, a similar procedure was adapted as described above, except a 4-pool Lorentzian model consisting of asymmetric MT, DS, NOE_-3.5ppm_, and NOE_-1.6ppm_ was fitted to the B0 corrected normalized spectra acquired at B1_sat_ = 1.4 μT. For Cr-based AREX we used *Δω* = 2 ppm (Fig 5A and 5E) and for PCr-based AREX, *Δω* = 2.64 ppm (Fig 5B and 5F). A linear correction for B1 was applied to the quantitative CEST maps using the ratio of the actual B1 value to the expected value in a voxel-by-voxel manner.

For Glu-CEST acquisition, we used B1_sat_ = 5.9 μT with 1000 ms saturation duration. For species such as fast-exchanging amine protons of glutamate, higher B1_sat_ amplitudes are required to increase the labeling efficiency. Since the baseline MT effect and the DS also increase with B1_sat_ amplitude, a shorter saturation duration is usually adopted to reduce the spillover dilution (Fig 1) [61]. Increase in DS of bulk water also confounds the CEST measurement and leads to loss of signal to noise ratio in metabolite specific CEST signal [62,63]. Additionally, for fast exchanging species, non-steady state based AREX calculations are roughly insensitive to T1 and T2 relaxations [42].

**Fig 6:**
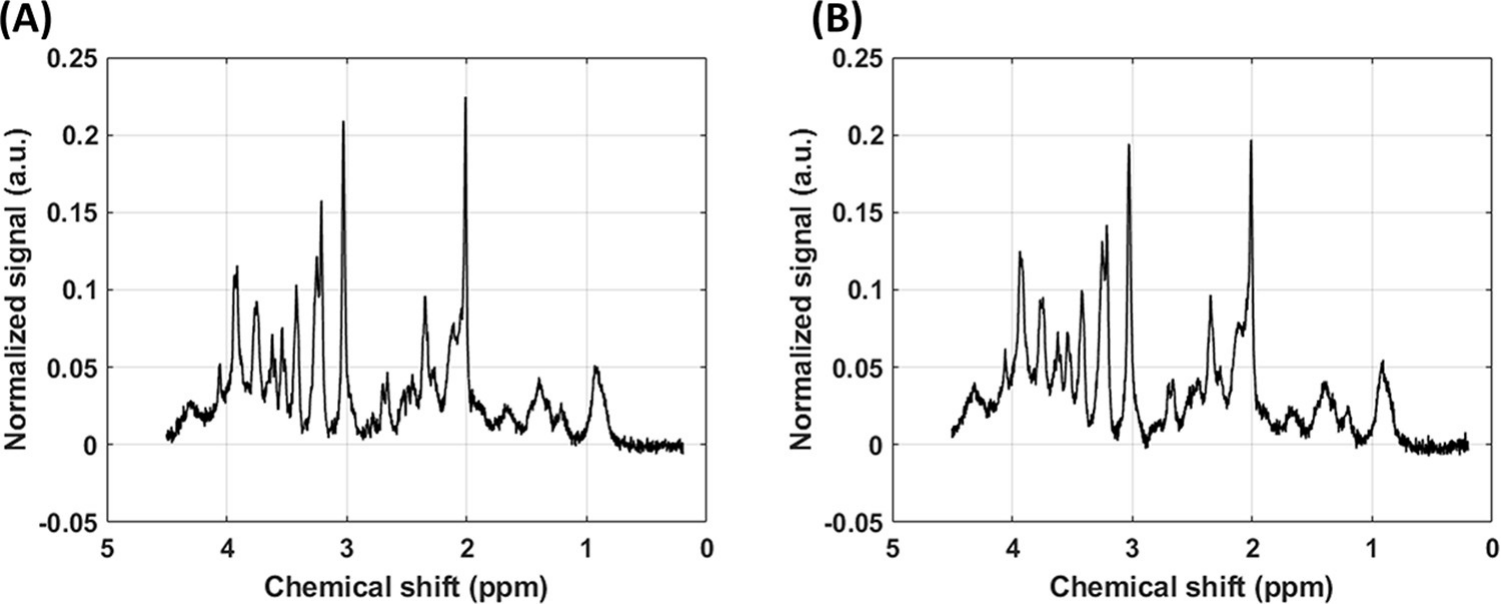
A representative single voxel ^1^H-MRS spectra from the right hippocampus of a (A) WT (control) and (B) ARTE10 animal.

Single-voxel based ^1^H-MRS absolute metabolite quantification was performed with the LCModel [64]. Before quantification, the raw water suppressed spectra were inspected for poor water suppression, lipid contamination, and motion artefacts. The simulated basis-set included spectra for alanine (Ala), aspartate (Asp), creatine (Cr), phosphocreatine (PCr), γ-aminobutyric acid (GABA), glucose (Glc), glutamine (Gln), glutamate (Glu), glutathione (GSH), glycerophosphpcholine (GPC), phosphocholine (PCh), myo-inositol (mIns), lactate (Lac), N-acetyl aspartate (NAA), N-acetylaspartylglutamate (NAAG), scyllo-inositol (Scyllo) and taurine (Tau). Absolute quantification used the unsuppressed water spectra as a reference. Corrections for GM, WM, and CSF tissue volume fractions and T1 relaxation of water were included in absolute metabolite quantification. The reliability of the measured metabolite concentration was estimated by Cramer-Rao lower bounds (CRLB) and only those metabolites with CRLB < 15% were selected for further analysis. The metabolite concentrations are reported in μmol/g. In vivo ^1^H-MRS spectra from representative WT and ARTE10 animals are shown in Fig 6.

### Statistical analysis

To investigate group differences in multiple CEST based contrasts and longitudinal relaxation time (qT1) in 1) region of the hippocampus overlapping with the MRS based voxel, and 2) tissue occupied by the MRS voxel, we applied independent *T*-test with general linear model (GLM). Benjamini-Hochberg false discovery rate (FDR) correction accounted for multiple comparison with *p*_*FDR*_ < 0.05 used as a significance criterion. The group differences in absolute metabolite concentration levels were investigated by independent *T*-tests with general linear model (GLM) and the same *p*_*FDR*_ < 0.05 significance criterion. Partial correlation analyses evaluated the relationships between ^1^H-MRS based neurochemical concentration levels and CEST derived parametric measures in the MRS voxel region. All statistical analyses were performed in SPSS (IBM, SPSS Version 28).

## Results

### Effect of synaptic deficit on the cerebral metabolites

Quantitative qT1 maps acquired from representative WT and ARTE10 animals highlight variation in T1 relaxation time across multiple tissue type (Fig 4A and 4E). Glu-AREX contrast, indicating tissue and pathology specific spatial distribution of glutamate is illustrated in Fig 4B and 4F. The normalized and corrected Z-spectra from the hippocampus ROI are presented in Fig 4C and 4D, while Fig 4G shows the Glu-AREX response spectra from the same ROI. Cr-AREX maps and PCr-AREX maps of representative WT and ARTE10 animals indicate tissue and pathology specific spatial distributions of Cr and PCr metabolites (Fig 5A, 5B, 5E and 5F, respectively). Normalized and corrected Z-spectra from the hippocampus ROI are shown in Fig 5C and 5D., while the Cr- and PCr-AREX response spectra from the same ROI are presented in Fig 5G.

Multiple CEST-based contrasts indicated significant group differences between ARTE10 and WT animals in the selected right hippocampus region (Table 2) and the regions within the MRS voxel (Supporting Information). ARTE10 exhibited significantly lower AREX-based Glu contrast (*p*_*FDR*_ < 0.05; Cohen’s *d* = −1.31, CI = (−2.672, 0.059)), PCr contrast (*p*_*FDR*_ < 0.05; Cohen’s *d* = −1.78, CI = (−3.253, −0.321)) and Cr contrast (*p*_*FDR*_ < 0.05; Cohen’s *d* = −1.08, CI = (−2.412, −0.243)). Single voxel MRS also yielded significant group differences between ARTE10 and WT animals (Table 3). ARTE10 animals exhibited significantly lower concentration levels of Glu (*p*_*FDR*_ *<* 0.05; Cohen’s *d* = −1.15, CI = (−2.75, 0.449)) and Glu/tCr ratio (*p*_*FDR*_ < 0.05; Cohen’s *d* = −3.072, CI = (−5.26, −0.885)). Total creatine (p_FDR_ < 0.05; Cohen’s *d* = 1.488, CI = (−0.186, 3.162)) and GSH (*p*_*FDR*_ < 0.05; Cohen’s *d* = 1.336, CI = (−0.302, 2.975)) concentration levels were significantly higher in ARTE10 animals, compared to WT counterparts.

**Table 2:** Comparison of neurometabolite weighting in the right hippocampus measured by CEST (N = 10 per group).

Metabolites weighting	Mean difference WT-ARTE10	*p*_*FDR*_	Effect size (Cohen’s d)	95% Confidence Interval
AREX-Glu (s^-1^)	0.06	0.011*	−1.31	−2.672, 0.059
AREX-PCr (s^-1^)	0.01	0.013*	−1.78	−3.253, −0.321
AREX-Cr (s^-1^)	0.01	0.03*	−1.08	−2.412, −0.243
qT1 (ms)	−35.37	0.18	0.59	−0.303, 1.49

Abbreviations: N = number of animals, AREX = apparent exchange dependent relaxation, Glu: Glutamate, PCr: Phosphocreatine, Cr: Creatine, qT1: Quantitative T1. Analysis adjusted for the hippocampus region of interest volume.

*p values were significant after a false-discovery rate (FDR) correction for multiple comparison *at p*_*FDR*_ < 0.05 using Benjamini-Hochberg criterion (α = 0.05).

**Table 3:** Comparison of neurometabolite concentration in the right hippocampus measured by ^1^H-MRS (N = 7 per group).

Metabolites [μmol/g]	Mean difference WT-ART10	*p*_*FDR*_	Effect size (Cohen’s d)	95% Confidence Interval
Glu	0.598	0.031*	-1.15	-2.75–0.449
Gln	-0.035	0.697	0.127	-1.356–1.61
Glx	0.563	0.028*	-1.217	-2.83–0.396
PCr	-0.612	0.002*	2.416	0.468–4.365
Cr	0.109	0.359	-0.565	-2.076–0.946
tCr	-0.503	0.033*	1.488	-0.186–3.162
GSH	-0.069	0.032*	1.336	-0.302–2.975
Glu/tCr	0.126	0.00015*	-3.072	-5.26 –-0.885
Glx/tCr	0.140	0.00004*	-3.787	-6.263 –-1.311

Analysis adjusted for water linewidth (Hz).

*p values were significant after a false-discovery rate (FDR) correction for multiple comparison *at p*_*FDR*_ < 0.05 using Benjamini-Hochberg criterion (α = 0.05).

### Associations between MRS measures and CEST-based contrasts

Table 4 shows the Pearson correlations between the regional metabolite concentration levels and mean values for CEST contrasts extracted from the MRS voxel located in the right hippocampus. MRS-based Glu/tCr ratio exhibited strong positive associations with AREX based Glu (*r* = 0.62, *p* < 0.05), Cr (*r* = 0.58, *p* < 0.05) and PCr (*r* = 0.58, *p* < 0.05) in the right hippocampus region. MRS Glu and "glutamate + glutamine” (Glx) were not significantly associated with AREX-based parameters. These linear correlation coefficient values represent the amount of variance that each parameter shares with the other.

**Table 4:** Partial correlation between MRS derived neurometabolite concentrations and CEST derived AREX parameters (N = 7 per group).

Metabolites [μmol/g]	AREX (Glu)		AREX (Cr)		AREX (PCr)	
	*r*	*p*	*r*	*p*	*r*	p
Glu	0.475	0.08^‡^	0.432	0.12	0.381	0.18
Glx	0.488	0.07^‡^	0.435	0.12	0.383	0.18
Glu/tCr	0.620	**0.02** *	0.583	0.03*	0.583	0.03*
Glx/tCr	0.623	**0.02** *	0.584	0.03*	0.598	0.02*

*Denotes *p* < 0.05

^‡^ denotes the association is not significant but trending. *p* values in bold font were significant after a false-discovery rate (FDR) correction for multiple comparison *p*_*FDR*_ < 0.05 using Benjamini-Hochberg criterion (α = 0.05).

## Discussion

We applied multi-modal MR to map the metabolic variations in the hippocampus of the animal model of AD and used high spatial-resolution, as well as low and high saturation power (B1_sat_) to obtain Cr-weighted and Glu-weighted CEST contrasts, respectively. Using Lorentzian fitting and qT1, we applied AREX correction in CEST contrast quantification to reduce the effects of spillover, symmetric/asymmetric MT, and T1 longitudinal relaxation. We performed absolute quantification of multiple cerebral metabolites in the right hippocampus region of the brain by using highly resolved short echo time spectra, qT1, and tissue volume fractions. Multiple CEST-based contrasts and regional MRS based metabolite concentration assessments allowed us to probe the synaptic density deficit in the ARTE10 animal model of AD pathology.

This multi-modal approach showed a significant decrease in AREX-based Glu and Cr contrasts in the hippocampus region of ARTE10 animals at 10 months of age. In the ARTE10 group, significant alteration in MRS-derived metabolites of neurotransmission and energy metabolism was also observed. We further examined the relationship between the regional neurochemistry and CEST-based contrasts and found significant association between Glu/tCr ratio and Glu-weighted CEST contrast in the hippocampus.

Glu is a primary excitatory neurotransmitter and plays an important role in neuroplasticity [65,66]. MR-based in vivo Glu quantification has been proposed as a surrogate for synaptic density assessment [22,23,67]. Single voxel MRS and PET based studies have demonstrated pathology specific associations between Glu/tCr ratio and PET tracer “^11^C-UCB-J” [68]. Multiple MRS-based studies have also reported reduced Glu levels in the hippocampus at the later stages of AD [69,70]. In conjunction with ^1^H-MRS and/or immunohistochemistry, multiple preclinical in vivo studies have demonstrated the efficacy of Glu-weighted CEST in animal models of neurodegeneration [26–34]. Crescenzi et al., 2018 [32] used Glu-CEST and histological evidence to demonstrate the association between Tau pathology mediated alteration in Glu-CEST contrast and synaptophysin based immunohistochemistry stains. Similarly, other preclinical studies on neurodegeneration also demonstrated varying degree of association between Glu-CEST contrast and MRS derived Glu/tCr ratio [27] and ELISA-based synaptophysin assessment [71].

In this study, for Glu-weighted CEST imaging we opted for a B1 saturation power of 5.9 μT to improve signal specificity for glutamate and to reduce MT baseline effect [42] and 1000 ms saturation pulse duration to achieve a stable signal. Strong DS and MT baseline effects due to high B1_sat_ and longer pulse duration were mitigated using Lorentzian fitting scheme. Additionally, B0 and B1 inhomogeneity corrections were made to improve signal specificity [22]. Our region of interest (ROI) analysis on Glu-CEST based results showed significant group differences between ARTE10 and WT animals in the hippocampus. The MRS-based results also showed significant reduction in Glu concentration levels in ARTE10 relative to WT animals. We also observed a strong positive association between the MRS-based Glu/tCr ratio and Glu-CEST based AREX parameter. However, a direct association between the MRS-derived Glu and Glu-AREX was not significant.

The CEST mechanisms such as chemical shift and variable exchange rate filter can provide a moderate to high level of metabolite specific signal contribution [22]. However, for Glu-CEST, lack of characteristic Glu peak makes the Glu signal quantification prone to contamination from creatine, GABA, aspartate, and amine protons [22,33,42]. Recent studies using tissue homogenates suggest that mobile protein species at 3.0 ppm significantly contribute to Glu-CEST signal [42]. Such non-specific signal contamination is much stronger at low B1 saturation power and inhomogeneous B1 presents as an additional factor in Glu-CEST signal non-specificity [72]. In this study, we used high B1 saturation power to enhance Glu signal contribution and double angle technique to correct for B1 transmit inhomogeneity. Despite these steps, the lack of association between Glu and Glu-AREX could be due to the limited sample size and/or non-specific signal contribution from other exchanging metabolites that resonate at neighboring frequencies [22]. Pathology mediated changes in regional pH, mobile proteins, and temperature can also influence Glu-CEST signal quantification [33,43].

The results of this study also suggest the involvement of multiple competing and compensating mechanisms associated with synaptic deficit. Our MRS-based results suggest an increase in creatine levels and GSH levels in ARTE10 animals, compared to WT group. Changes in ^1^H-MRS-based tCr and GSH may suggest distruption in energy metabolism [15] and/or the involvement of compensatory mechanism associated with oxidation-reduction reaction [73]. Our Cr-CEST based analysis, on the other hand, suggested a significant increase in Cr-AREX in WT, relative to the ARTE10 animals. Additionally, Glu/tCr ratio showed positive association with Cr-AREX indices. This contradiction between MRS-based tCr and CEST-based Cr assessments suggests the influence of confounding factors in Cr-CEST quantification [21]. Recently, it has been reported that guanidinum protons from arginine side chains in mobile proteins have a significant contribution to Cr-CEST signal in vitro [21,74]. This non-specific signal contribution from mobile proteins resonating at 2.0 ppm could account for the observed disparity between our MRS-based tCr and Cr-CEST assessments. Moreover, a recent study reported a strong association between ^1^H-MRS-based tCr concentration levels and Cr-CEST-based parameters [75] and a subsequent study on murine models of neurodegeneration attributed the Cr-CEST changes to pathology specific changes in cerebral pH [21]. In contrast to our findings, these studies did not report changes in ^1^H-MRS based creatine levels in AD animals. It is important to note that while these studies corrected their CEST-based quantification for potential T1 relaxation variations, such corrections along with tissue volume fraction contributions were not considered in their ^1^H-MRS based quantification. Additionally, one should interpret results from in vitro studies with caution, as the absence of tissue compartmentalization in homogenates may impact the exchange rate and solvent accessibility of exchanging protons, introducing potential biases in CEST signal specificity[22].

Due to the highly resolved nature of our acquired ^1^H-MRS spectra, we were able to separately quantify PCr and Cr concentration levels. Group analysis on individual creatine components revealed elevated PCr in the hippocampus and no significant changes in the Cr levels. These findings are in agreement with in-vivo ^31^P-MRS study on early AD pathology [76]. Neuroinflammation has been suggested as an important factor in altering cerebral pH leading to dysfunctional cellular metabolism [77,78]. However, our ^1^H-MRS based myo-inositol results (not shown) were not significantly different between the groups, suggesting the involvement of additional contributing factors in pH alteration [21]. There is some evidence supporting the upregulation of PCr as a mechanism against glutamate excitotoxicity [79]. Since PCr regulates the uptake of Glu via synaptic vesicles [80], elevated levels of PCr, as shown in our study, may indicate pathology mediated underutilization of adenosine triphosphate (ATP) [81] due to decreased levels of glucose uptake in hippocampus [82] or as a compensatory mechanism to regulate Glu excitotoxicity [76]. Further research is warranted to elucidate the role of energy metabolism in Aβ induced synaptic deficit and synaptic dysfunction.

This study has some limitations. Due to the exploratory nature of the work, we only scanned male mice. For ^1^H-MRS scans, we used a subset of animals from each group (N = 7/group), and only scanned the right hippocampus region. Due to the region-specific impact of AD, other brain regions certainly merit further investigation. In addition, for CEST-based analyses, the sample size was relatively small (N = 10 per group) and therefore, these findings should be considered with caution. The study lacks validation of in vivo metabolite weighted CEST contrasts through immunohistochemistry (IHC), synaptic vesicle glycoprotein 2A (SV2A) in vivo PET, or SV2A autoradiography. In future research, a robust validation strategy should involve mapping either by using IHC stains of pre- and postsynaptic density markers or in vivo SV2A PET with high-resolution in vivo CEST contrasts. Such multi-modal in vivo/ex vivo correlations promise a more detailed understanding of biochemical mechanisms during the pathology’s progression. Future studies should prioritize a larger sample size and incorporate a longitudinal design to capture the dynamic evolution of various metabolites throughout the disease trajectory. Furthermore, the inclusion of female mice would enable the assessment of potential sex-related effects, contributing to a more comprehensive understanding of the observed phenomena.

In this study we used multi-modal MR to map and quantify alterations in neurometabolites in ARTE10 animal model of AD. Due to the unique characteristics of this AD model, we were able to probe the variations in cerebral metabolites associated with synaptic degradation. Given the molecular specificity of quantitative MR based metabolite imaging, such a multi-modal quantitative MR method has the potential to detect abnormal cerebral molecular changes possibly before pathology associated microstructural and macrostructural alterations in brain and thus may serve as an early biomarker in neuropathology.

## Supporting information

S1 Data (CSV)

S1 File Supporting information- contains all the supporting tables. (DOCX)

## References

[1] Hardy J. and Selkoe D. J., "The amyloid hypothesis of Alzheimer’s disease: progress and problems on the road to therapeutics," *science*, vol. 297, no. 5580, pp. 353–356, 2002. doi: 10.1126/science.1072994 12130773

[2] Tsai J., Grutzendler J., Duff K., and Gan W.-B., "Fibrillar amyloid deposition leads to local synaptic abnormalities and breakage of neuronal branches," *Nature neuroscience*, vol. 7, no. 11, pp. 1181–1183, 2004. doi: 10.1038/nn1335 15475950

[3] Selkoe D. J., "Alzheimer’s disease is a synaptic failure," *Science*, vol. 298, no. 5594, pp. 789–791, 2002. doi: 10.1126/science.1074069 12399581

[4] Imbriani P., Schirinzi T., Meringolo M., Mercuri N. B., and Pisani A., "Centrality of early synaptopathy in Parkinson’s disease," *Frontiers in neurology*, vol. 9, p. 103, 2018. doi: 10.3389/fneur.2018.00103 29545770 PMC5837972

[5] Colom-Cadena M. et al., "The clinical promise of biomarkers of synapse damage or loss in Alzheimer’s disease," *Alzheimer’s research & therapy*, vol. 12, pp. 1–12, 2020. doi: 10.1186/s13195-020-00588-4 32122400 PMC7053087

[6] Li J.-Y., Plomann M., and Brundin P., "Huntington’s disease: a synaptopathy?," *Trends in molecular medicine*, vol. 9, no. 10, pp. 414–420, 2003. doi: 10.1016/j.molmed.2003.08.006 14557053

[7] Ettcheto M. et al., "Pharmacological strategies to improve dendritic spines in Alzheimer’s disease," *Journal of Alzheimer’s Disease*, vol. 82, no. s1, pp. S91–S107, 2021. doi: 10.3233/JAD-201106 33325386 PMC9853464

[8] Jacobsen J. S. et al., "Early-onset behavioral and synaptic deficits in a mouse model of Alzheimer’s disease," *Proceedings of the National Academy of Sciences*, vol. 103, no. 13, pp. 5161–5166, 2006. 10.1073/pnas.0600948103 PMC1405622 16549764

[9] Henstridge C. M., Hyman B. T., and Spires-Jones T. L., "Beyond the neuron–cellular interactions early in Alzheimer disease pathogenesis," *Nature Reviews Neuroscience*, vol. 20, no. 2, pp. 94–108, 2019. doi: 10.1038/s41583-018-0113-1 30643230 PMC6545070

[10] Oeltzschner G. et al., "Neurometabolites and associations with cognitive deficits in mild cognitive impairment: a magnetic resonance spectroscopy study at 7 Tesla," *Neurobiology of aging*, vol. 73, pp. 211–218, 2019. 30390554 10.1016/j.neurobiolaging.2018.09.027 PMC6294473

[11] Wang W., Zhao F., Ma X., Perry G., and Zhu X., "Mitochondria dysfunction in the pathogenesis of Alzheimer’s disease: Recent advances," *Mol Neurodegener*, vol. 15, pp. 1–22, 2020. 32471464 10.1186/s13024-020-00376-6 PMC7257174

[12] Cai Q. and Tammineni P., "Mitochondrial aspects of synaptic dysfunction in Alzheimer’s disease," *Journal of Alzheimer’s disease*, vol. 57, no. 4, pp. 1087–1103, 2017. doi: 10.3233/JAD-160726 27767992 PMC5398949

[13] Swerdlow R. H., Burns J. M., and Khan S. M., "The Alzheimer’s disease mitochondrial cascade hypothesis: progress and perspectives," *Biochimica et Biophysica Acta (BBA)-Molecular Basis of Disease*, vol. 1842, no. 8, pp. 1219–1231, 2014. doi: 10.1016/j.bbadis.2013.09.010 24071439 PMC3962811

[14] Guo L., Tian J., and Du H., "Mitochondrial dysfunction and synaptic transmission failure in Alzheimer’s disease," *Journal of Alzheimer’s Disease*, vol. 57, no. 4, pp. 1071–1086, 2017. doi: 10.3233/JAD-160702 27662318 PMC5605817

[15] Bürklen T. S. et al., "The creatine kinase/creatine connection to alzheimer’s disease: CK Inactivation, APP-CK complexes and focal creatine deposits," *Journal of Biomedicine and Biotechnology*, vol. 2006, 2006. doi: 10.1155/JBB/2006/35936 17047305 PMC1510941

[16] Vos M., Lauwers E., and Verstreken P., "Synaptic mitochondria in synaptic transmission and organization of vesicle pools in health and disease," *Frontiers in synaptic neuroscience*, vol. 2, p. 139, 2010. doi: 10.3389/fnsyn.2010.00139 21423525 PMC3059669

[17] Chen M.-K. et al., "Assessing synaptic density in Alzheimer disease with synaptic vesicle glycoprotein 2A positron emission tomographic imaging," *JAMA neurology*, vol. 75, no. 10, pp. 1215–1224, 2018. doi: 10.1001/jamaneurol.2018.1836 30014145 PMC6233853

[18] Vanhaute H. et al., "In vivo synaptic density loss is related to tau deposition in amnestic mild cognitive impairment," *Neurology*, vol. 95, no. 5, pp. e545–e553, 2020. doi: 10.1212/WNL.0000000000009818 32493717

[19] Coomans E. M. et al., "In vivo tau pathology is associated with synaptic loss and altered synaptic function," *Alzheimer’s research & therapy*, vol. 13, no. 1, pp. 1–13, 2021. doi: 10.1186/s13195-021-00772-0 33546722 PMC7866464

[20] Becker G., Dammicco S., Bahri M. A., and Salmon E., "The rise of synaptic density PET imaging," *Molecules*, vol. 25, no. 10, p. 2303, 2020. doi: 10.3390/molecules25102303 32422902 PMC7288098

[21] Chen L. et al., "Early detection of Alzheimer’s disease using creatine chemical exchange saturation transfer magnetic resonance imaging," *NeuroImage*, vol. 236, p. 118071, 2021. doi: 10.1016/j.neuroimage.2021.118071 33878375 PMC8321389

[22] Cember A. T., Nanga R. P. R., and Reddy R., "Glutamate‐weighted CEST (gluCEST) imaging for mapping neurometabolism: An update on the state of the art and emerging findings from in vivo applications," *NMR in Biomedicine*, p. e4780, 2022. doi: 10.1002/nbm.4780 35642353

[23] Serrano M. E., Kim E., Petrinovic M. M., Turkheimer F., and Cash D., "Imaging synaptic density: the next holy grail of neuroscience?," *Frontiers in neuroscience*, vol. 16, p. 796129, 2022. doi: 10.3389/fnins.2022.796129 35401097 PMC8990757

[24] Agarwal N. and Renshaw P., "Proton MR spectroscopy–detectable major neurotransmitters of the brain: Biology and possible clinical applications," *American journal of neuroradiology*, vol. 33, no. 4, pp. 595–602, 2012. doi: 10.3174/ajnr.A2587 22207303 PMC4627491

[25] Willuweit A. et al., "Comparison of the amyloid load in the brains of two transgenic Alzheimer’s disease mouse models quantified by florbetaben positron emission tomography," *Frontiers in neuroscience*, vol. 15, p. 699926, 2021. doi: 10.3389/fnins.2021.699926 34671235 PMC8520975

[26] Chen Y. et al., "Imaging of glutamate in brain abscess using GLUCEST at 7T," *Radiology of Infectious Diseases*, vol. 5, no. 4, pp. 148–153, 2018.

[27] Haris M. et al., "Imaging of glutamate neurotransmitter alterations in Alzheimer’s disease," *NMR in biomedicine*, vol. 26, no. 4, pp. 386–391, 2013. doi: 10.1002/nbm.2875 23045158 PMC3556355

[28] Pépin J., Jego P., Valette J., Bonvento G., Flament J., and Roses F., "Imaging of neuronal compartment using gluCEST method," in *International Society for Magnetic Resonance in Medicine*, 2017.

[29] Pépin J. et al., "In vivo imaging of brain glutamate defects in a knock-in mouse model of Huntington’s disease," *Neuroimage*, vol. 139, pp. 53–64, 2016. doi: 10.1016/j.neuroimage.2016.06.023 27318215

[30] Crescenzi R. et al., "In vivo measurement of glutamate loss is associated with synapse loss in a mouse model of tauopathy," *Neuroimage*, vol. 101, pp. 185–192, 2014. doi: 10.1016/j.neuroimage.2014.06.067 25003815 PMC4303593

[31] Bagga P., Chugani A. N., Varadarajan K. S., and Patel A. B., "In vivo NMR studies of regional cerebral energetics in MPTP model of P arkinson’s disease: recovery of cerebral metabolism with acute levodopa treatment," *Journal of neurochemistry*, vol. 127, no. 3, pp. 365–377, 2013. doi: 10.1111/jnc.12407 23957451

[32] Crescenzi R. et al., "Longitudinal imaging reveals subhippocampal dynamics in glutamate levels associated with histopathologic events in a mouse model of tauopathy and healthy mice," *Hippocampus*, vol. 27, no. 3, pp. 285–302, 2017. doi: 10.1002/hipo.22693 27997993 PMC5396955

[33] Cai K. et al., "Magnetic resonance imaging of glutamate," *Nature medicine*, vol. 18, no. 2, pp. 302–306, 2012. doi: 10.1038/nm.2615 22270722 PMC3274604

[34] Chen Y. et al., "Magnetic resonance imaging of glutamate in neuroinflammation," *Radiology of Infectious Diseases*, vol. 3, no. 2, pp. 92–97, 2016.

[35] Möller H. E., "Considerations on gradual glutamate accumulation related to cognitive task performance," *Journal of Cerebral Blood Flow & Metabolism*, vol. 43, no. 3, pp. 476–478, 2023. doi: 10.1177/0271678X221139550 36369737 PMC9941861

[36] Takado Y. et al., "MRS-measured glutamate versus GABA reflects excitatory versus inhibitory neural activities in awake mice," *Journal of Cerebral Blood Flow & Metabolism*, vol. 42, no. 1, pp. 197–212, 2022. doi: 10.1177/0271678X211045449 34515548 PMC8721779

[37] Kauppinen R., Pirttilä T., Auriola S., and Williams S., "Compartmentation of cerebral glutamate in situ as detected by 1H/13C nmr," *Biochemical Journal*, vol. 298, no. 1, pp. 121–127, 1994. 7907470 10.1042/bj2980121 PMC1137991

[38] Najac C. et al., "Intracellular metabolites in the primate brain are primarily localized in long fibers rather than in cell bodies, as shown by diffusion-weighted magnetic resonance spectroscopy," *Neuroimage*, vol. 90, pp. 374–380, 2014. doi: 10.1016/j.neuroimage.2013.12.045 24382523

[39] Maddock R. J. and Buonocore M. H., "MR spectroscopic studies of the brain in psychiatric disorders," *Brain imaging in behavioral neuroscience*, pp. 199–251, 2012. doi: 10.1007/7854_2011_197 22294088

[40] Lalande J. et al., "1H NMR metabolomic signatures in five brain regions of the AbetaPPswe Tg2576 mouse model of Alzheimer’s disease at four ages," (in eng), *J Alzheimers Dis*, vol. 39, no. 1, pp. 121–43, 2014, doi: 10.3233/JAD-130023 24145382

[41] Bie C., van Zijl P., Xu J., Song X., and Yadav N. N., "Radiofrequency (RF) labeling strategies in chemical exchange saturation transfer (CEST) MRI," *NMR in Biomedicine*, p. e4944, 2023. 37002814 10.1002/nbm.4944 PMC10312378

[42] Cui J. and Zu Z., "Towards the molecular origin of glutamate CEST (GluCEST) imaging in rat brain," *Magnetic Resonance In Medicine*, vol. 83, no. 4, pp. 1405–1417, 2020. doi: 10.1002/mrm.28021 31691367

[43] Khlebnikov V., van der Kemp W. J., Hoogduin H., Klomp D. W., and Prompers J. J., "Analysis of chemical exchange saturation transfer contributions from brain metabolites to the Z-spectra at various field strengths and pH," *Scientific reports*, vol. 9, no. 1, p. 1089, 2019. doi: 10.1038/s41598-018-37295-y 30705355 PMC6355971

[44] Khlebnikov V. et al., "On the transmit field inhomogeneity correction of relaxation‐compensated amide and NOE CEST effects at 7 T," *NMR in Biomedicine*, vol. 30, no. 5, p. e3687, 2017. 28111824 10.1002/nbm.3687 PMC5412922

[45] Willuweit A. et al., "Early-onset and robust amyloid pathology in a new homozygous mouse model of Alzheimer’s disease," *PloS one*, vol. 4, no. 11, p. e7931, 2009. doi: 10.1371/journal.pone.0007931 19936202 PMC2775952

[46] Šišková Z. et al., "Dendritic structural degeneration is functionally linked to cellular hyperexcitability in a mouse model of Alzheimer’s disease," *Neuron*, vol. 84, no. 5, pp. 1023–1033, 2014. doi: 10.1016/j.neuron.2014.10.024 25456500

[47] Kim M., Gillen J., Landman B. A., Zhou J., and Van Zijl P. C., "Water saturation shift referencing (WASSR) for chemical exchange saturation transfer (CEST) experiments," *Magnetic Resonance in Medicine*: *An Official Journal of the International Society for Magnetic Resonance in Medicine*, vol. 61, no. 6, pp. 1441–1450, 2009. doi: 10.1002/mrm.21873 19358232 PMC2860191

[48] Stollberger R. and Wach P., "Imaging of the active B1 field in vivo," *Magnetic resonance in medicine*, vol. 35, no. 2, pp. 246–251, 1996. doi: 10.1002/mrm.1910350217 8622590

[49] Garwood M. and DelaBarre L., "The return of the frequency sweep: designing adiabatic pulses for contemporary NMR," *Journal of magnetic resonance*, vol. 153, no. 2, pp. 155–177, 2001. doi: 10.1006/jmre.2001.2340 11740891

[50] Slotboom J. and Bovée W., "Adiabatic slice‐selective rf pulses and a single‐shot adiabatic localization pulse sequence," *Concepts in Magnetic Resonance*, vol. 7, no. 3, pp. 193–217, 1995.

[51] Coupe P., Yger P., Prima S., Hellier P., Kervrann C., and Barillot C., "An optimized blockwise nonlocal means denoising filter for 3-D magnetic resonance images," *IEEE Trans Med Imaging*, vol. 27, no. 4, pp. 425–41, Apr 2008, doi: 10.1109/TMI.2007.906087 18390341 PMC2881565

[52] Tustison N. J. et al., "N4ITK: improved N3 bias correction," *IEEE Trans Med Imaging*, vol. 29, no. 6, pp. 1310–20, Jun 2010, doi: 10.1109/TMI.2010.2046908 20378467 PMC3071855

[53] Avants B. B., Tustison N. J., Wu J., Cook P. A., and Gee J. C., "An open source multivariate framework for n-tissue segmentation with evaluation on public data," *Neuroinformatics*, vol. 9, no. 4, pp. 381–400, 2011. doi: 10.1007/s12021-011-9109-y 21373993 PMC3297199

[54] Martinez P. et al., "Bassoon contributes to tau-seed propagation and neurotoxicity," *Nature neuroscience*, vol. 25, no. 12, pp. 1597–1607, 2022. doi: 10.1038/s41593-022-01191-6 36344699 PMC9708566

[55] Landheer K., Gajdošík M., and Juchem C., "A semi‐LASER, single‐voxel spectroscopic sequence with a minimal echo time of 20.1 ms in the human brain at 3 T," *NMR in Biomedicine*, vol. 33, no. 9, p. e4324, 2020. doi: 10.1002/nbm.4324 32557880

[56] Evans V. S. et al., "Optimization and repeatability of multipool chemical exchange saturation transfer MRI of the prostate at 3.0 T," *Journal of Magnetic Resonance Imaging*, vol. 50, no. 4, pp. 1238–1250, 2019. 30770603 10.1002/jmri.26690 PMC6767527

[57] Zu Z., "Toward more reliable measurements of NOE effects in CEST spectra at around− 1.6 ppm (NOE (− 1.6)) in rat brain," *Magnetic resonance in medicine*, vol. 81, no. 1, pp. 208–219, 2019. 30058128 10.1002/mrm.27370 PMC6258343

[58] Mennecke A. et al., "7 tricks for 7 T CEST: Improving the reproducibility of multipool evaluation provides insights into the effects of age and the early stages of Parkinson’s disease," *NMR in Biomedicine*, vol. 36, no. 6, p. e4717, 2023. doi: 10.1002/nbm.4717 35194865

[59] Zhang X. Y. et al., "Accuracy in the quantification of chemical exchange saturation transfer (CEST) and relayed nuclear Overhauser enhancement (rNOE) saturation transfer effects," *NMR in biomedicine*, vol. 30, no. 7, p. e3716, 2017. doi: 10.1002/nbm.3716 28272761 PMC5490367

[60] Hua J., Jones C. K., Blakeley J., Smith S. A., Van Zijl P. C., and Zhou J., "Quantitative description of the asymmetry in magnetization transfer effects around the water resonance in the human brain," *Magnetic Resonance in Medicine*: *An Official Journal of the International Society for Magnetic Resonance in Medicine*, vol. 58, no. 4, pp. 786–793, 2007. doi: 10.1002/mrm.21387 17899597 PMC3707117

[61] Zaiss M., Jin T., Kim S. G., and Gochberg D. F., "Theory of chemical exchange saturation transfer MRI in the context of different magnetic fields," *NMR in Biomedicine*, vol. 35, no. 11, p. e4789, 2022. doi: 10.1002/nbm.4789 35704180

[62] Hoefemann M., Döring A., Fichtner N. D., and Kreis R., "Combining chemical exchange saturation transfer and 1H magnetic resonance spectroscopy for simultaneous determination of metabolite concentrations and effects of magnetization exchange," *Magnetic resonance in medicine*, vol. 85, no. 4, pp. 1766–1782, 2021. doi: 10.1002/mrm.28574 33151011 PMC7821128

[63] Zu Z., Janve V. A., Xu J., Does M. D., Gore J. C., and Gochberg D. F., "A new method for detecting exchanging amide protons using chemical exchange rotation transfer," *Magnetic Resonance in Medicine*, vol. 69, no. 3, pp. 637–647, 2013. doi: 10.1002/mrm.24284 22505325 PMC3625661

[64] Provencher S. W., "Automatic quantitation of localized in vivo 1H spectra with LCModel," *NMR Biomed*, vol. 14, no. 4, pp. 260–4, Jun 2001, doi: 10.1002/nbm.698 11410943

[65] Erecinska M. and Silver I. A., "Metabolism and role of glutamate in mammalian brain," *Progress in neurobiology*, vol. 35, no. 4, pp. 245–96, 1990, doi: 10.1016/0301-0082(90)90013-7 1980745

[66] Danbolt N. C., "Glutamate uptake," *Progress in neurobiology*, vol. 65, no. 1, pp. 1–105, Sep 2001, doi: 10.1016/s0301-0082(00)00067-8 11369436

[67] Cox M. F., Hascup E. R., Bartke A., and Hascup K. N., "Friend or foe? Defining the role of glutamate in aging and Alzheimer’s disease," *Frontiers in Aging*, vol. 3, p. 929474, 2022. doi: 10.3389/fragi.2022.929474 35821835 PMC9261322

[68] Onwordi E. C. et al., "The relationship between synaptic density marker SV2A, glutamate and N-acetyl aspartate levels in healthy volunteers and schizophrenia: a multimodal PET and magnetic resonance spectroscopy brain imaging study," *Translational psychiatry*, vol. 11, no. 1, p. 393, 2021. doi: 10.1038/s41398-021-01515-3 34282130 PMC8290006

[69] Fayed N., Modrego P. J., Rojas-Salinas G., and Aguilar K., "Brain glutamate levels are decreased in Alzheimer’s disease: a magnetic resonance spectroscopy study," *American Journal of Alzheimer’s Disease & Other Dementias* *®*, vol. 26, no. 6, pp. 450–456, 2011. doi: 10.1177/1533317511421780 21921084 PMC10845671

[70] Lin A. P, Shic F., Enriquez C., and Ross B. D., "Reduced glutamate neurotransmission in patients with Alzheimer’s disease–an in vivo 13 C magnetic resonance spectroscopy study," *Magnetic Resonance Materials in Physics*, *Biology and Medicine*, vol. 16, pp. 29–42, 2003. doi: 10.1007/s10334-003-0004-x 12695884

[71] Igarashi H. et al., "Longitudinal GluCEST MRI changes and cerebral blood flow in 5xFAD mice," *Contrast Media & Molecular Imaging*, vol. 2020, pp. 1–12, 2020. doi: 10.1155/2020/8831936 33304204 PMC7714610

[72] Cember A. T., Hariharan H., Kumar D., Nanga R. P., and Reddy R., "Improved method for post‐processing correction of B1 inhomogeneity in glutamate‐weighted CEST images of the human brain," *NMR in Biomedicine*, vol. 34, no. 6, p. e4503, 2021. doi: 10.1002/nbm.4503 33749037

[73] Emir U. E. et al., "Noninvasive quantification of ascorbate and glutathione concentration in the elderly human brain," *NMR in Biomedicine*, vol. 24, no. 7, pp. 888–894, 2011. doi: 10.1002/nbm.1646 21834011 PMC3118919

[74] Zhang X. Y. et al., "Assignment of the molecular origins of CEST signals at 2 ppm in rat brain," *Magnetic resonance in medicine*, vol. 78, no. 3, pp. 881–887, 2017. 28653349 10.1002/mrm.26802 PMC5561473

[75] Chen L. et al., "Investigation of the contribution of total creatine to the CEST Z‐spectrum of brain using a knockout mouse model," *NMR in biomedicine*, vol. 30, no. 12, p. e3834, 2017. 10.1002/nbm.3834 PMC5685917 28961344

[76] Rijpma A., van der Graaf M., Meulenbroek O., Rikkert M. G. O., and Heerschap A., "Altered brain high-energy phosphate metabolism in mild Alzheimer’s disease: A 3-dimensional 31P MR spectroscopic imaging study," *NeuroImage*: *Clinical*, vol. 18, pp. 254–261, 2018. doi: 10.1016/j.nicl.2018.01.031 29876246 PMC5987799

[77] Schwartz L., Peres S., Jolicoeur M., and da Veiga Moreira J., "Cancer and Alzheimer’s disease: intracellular pH scales the metabolic disorders," *Biogerontology*, vol. 21, pp. 683–694, 2020. doi: 10.1007/s10522-020-09888-6 32617766

[78] Eikelenboom P., Van Exel E., Hoozemans J. J., Veerhuis R., Rozemuller A. J., and Van Gool W. A., "Neuroinflammation–an early event in both the history and pathogenesis of Alzheimer’s disease," *Neurodegenerative Diseases*, vol. 7, no. 1–3, pp. 38–41, 2010. doi: 10.1159/000283480 20160456

[79] Bender A.* et al., "Creatine supplementation lowers brain glutamate levels in Huntington’s disease," *Journal of neurology*, vol. 252, pp. 36–41, 2005. doi: 10.1007/s00415-005-0595-4 15672208

[80] Xu C. J., Klunk W. E., Kanfer J. N., Xiong Q., Miller G., and Pettegrew J. W., "Phosphocreatine-dependent glutamate uptake by synaptic vesicles: a comparison with atp-dependent glutamate uptake," *Journal of Biological Chemistry*, vol. 271, no. 23, pp. 13435–13440, 1996. 8662761 10.1074/jbc.271.23.13435

[81] Forester B. P. et al., "Age‐related changes in brain energetics and phospholipid metabolism," *NMR in Biomedicine*, vol. 23, no. 3, pp. 242–250, 2010. doi: 10.1002/nbm.1444 19908224

[82] Parasoglou P. et al., "Phosphorus metabolism in the brain of cognitively normal midlife individuals at risk for Alzheimer’s disease," *Neuroimage*: *Reports*, vol. 2, no. 4, p. 100121, 2022. doi: 10.1016/j.ynirp.2022.100121 36532654 PMC9757821

