# Supplementary material for: Estimating the synaptic density deficit in Alzheimer’s disease using multi-contrast CEST imaging
Source: PLoS One. 2024 Mar 14;19(3):e0299961. doi: 10.1371/journal.pone.0299961 (PMC10939256; doi:10.1371/journal.pone.0299961)
Supplement: S1 File — (DOCX) [file pone.0299961.s002.docx]

**S1 Table: Comparison of neurometabolite weighting in the right hippocampus measured by CEST (N=10 per group).**

| **Metabolites weighting** | **Mean difference**  **WT-ARTE10** | ***p_FDR_*** | **Effect size**  **(Cohen’s d)** | **95% Confidence Interval** |
| --- | --- | --- | --- | --- |
| AREX-Glu (s^-1^) | 0.07 | 0.014* | −1.38 | −2.364, −0.411 |
| AREX-PCr (s^-1^) | 0.01 | 0.011* | −1.37 | −2.346, −0.398 |
| AREX-Cr (s^-1^) | 0.02 | 0.016* | −1.17 | −2.123, −0.225 |
| qT1 (ms) | −40.12 | 0.23 | 0.59 | −0.302, 1.49 |

Abbreviations: N= number of animals, AREX= apparent exchange-dependent relaxation, Glu: glutamate, PCr: phosphocreatine, Cr: creatine, qT1: Quantitative T1. Analysis adjusted for MRS voxel volume.

**p* values were significant after a false-discovery rate (FDR) correction for multiple comparison *at p_FDR_* < 0.05 using Benjamini-Hochberg criterion (α=0.05).
